# Trauma resuscitation requiring massive transfusion: a descriptive analysis of the role of ratio and time

**DOI:** 10.1186/s13017-015-0028-3

**Published:** 2015-08-14

**Authors:** Ruben Peralta, Adarsh Vijay, Ayman El-Menyar, Rafael Consunji, Husham Abdelrahman, Ashok Parchani, Ibrahim Afifi, Ahmad Zarour, Hassan Al-Thani, Rifat Latifi

**Affiliations:** Trauma Surgery Section, Hamad Trauma Center, Hamad General Hospital, Doha, Qatar; Clinical Research, Trauma Surgery Section, Hamad General Hospital, HMC, PO Box 3050, Doha, Qatar; Clinical Medicine, Weill Cornell Medical College, Doha, Qatar; Department of Surgery, University of Arizona, Tucson, AZ USA

**Keywords:** Trauma, Transfusion ratio, Massive transfusion protocol, Outcome

## Abstract

**Objective:**

We aimed to evaluate whether early administration of high plasma to red blood cells ratios influences outcomes in injured patients who received massive transfusion protocol (MTP).

**Methods:**

A retrospective analysis was conducted at the only level 1 national trauma center in Qatar for all adult patients(≥18 years old) who received MTP (≥10 units) of packed red blood cell (PRBC) during the initial 24 h post traumatic injury. Data were analyzed with respect to FFB:PRBC ratio [(high ≥ 1:1.5) (HMTP) *vs.* (low < 1:1.5) (LMTP)] given at the first 4 h post-injury and also between (>4 and 24 h). Mortality, multiorgan failure (MOF) and infectious complications were studied as well.

**Results:**

During the study period, a total of 4864 trauma patients were admitted to the hospital, 1.6 % (n = 77) of them met the inclusion criteria. Both groups were comparable with respect to initial pH, international normalized ratio, injury severity score, revised trauma score and development of infectious complications. However, HMTP was associated with lower crude mortality (41.9 *vs.* 78.3 %, *p* = 0.001) and lower rate of MOF (48.4 *vs.* 87.0 %, *p* = 0.001). The number of deaths was 3 times higher in LMTP in comparison to HMTP within the first 30 days (36 *vs.* 13 cases). The majority of deaths occurred within the first 24 h (80.5 % in LMTP and 69 % in HMTP) and particularly within the first 6 h (55 *vs.* 46 %).

**Conclusions:**

Aggressive attainment of high FFP/PRBC ratios as early as 4 h post-injury can substantially improve outcomes in trauma patients.

## Background

In severe trauma patients, exsanguinating hemorrhage is the most common cause of early death, which occurs within the first few hours of hospital admission [1, 2]. Up to 30 % of severely injured patients are coagulopathic upon arrival to the emergency department [3]. Moreover, the coagulopathy status developed early post traumatic injury invariably complicates the massive blood transfusion and damage control resuscitation pattern and outcome. Prior reports of the military and civilian experience that advocated the use of higher ratios of fresh frozen plasma (FFP) to packed red blood cells (PRBCs) showed an improvement in survival of trauma patients [4, 5]. These studies have addressed the implications of early aggressive hemostatic resuscitation to control coagulopathy, which potentially reduces the mortality rate. However, some investigators suggested that transfusion of inappropriate volumes of plasma might result in a higher incidence of infection or transfusion-associated acute lung injury [6–8]. According to the current guidelines of hemostatic resuscitation, severe trauma patients should be supplemented with a considerable amount of blood products (PRBC, FFP and platelet) in an early and continuous manner [9]. However, there is no consensus on the optimal ratio of FFP to PRBC for trauma patients requiring massive transfusion protocol (MTP). Borgman et al. [4] proposed a predictive model of MTP in severe trauma patients. The authors suggested that a high ratio (FFP: RBC) (≥15 TASH score) was an independent predictor of survival; whereas, the low ratio (<15 TASH score) was correlated well with higher complication rates.

There is still ongoing debate to define the most appropriate time and ratio of MTP that should be given to patients undergoing hemostatic resuscitation. Specifically, the appropriate timing and resuscitation ratio during the early hours of severe trauma remain unclear. Herein, we aimed to evaluate whether early administration of high plasma ratios to RBC influences outcomes in trauma patients receiving massive blood transfusion.

## Methods

We conducted a retrospective analysis of the prospectively collected data of all adult trauma (≥18 years old) patients who received MTP. The study was conducted at the Hamad Trauma Center which is the only level 1 national trauma referral center in Qatar from January 2010 to December 2012. Blood bank records were used to identify patients who received more than 6 units PRBC. The records were then screened for subset of patients who received more than 10 units PRBC in the first 24 h. The medical records of patients were obtained to evaluate the ratio based on actual blood products transfused, the actual time of transfusion along with the details of other comorbid conditions, complications and outcome. All patients who died on arrival were excluded. We defined MTP as the infusion of ≥10 units of PRBC over the initial 24 h post-injury [10]. Injury Severity Scores (ISS) and Revised Trauma Scores (RTS) were collected from the hospital trauma database for all cases. Based on FFP: PRBC ratio at 4 h post-injury; patients were categorized into two groups i.e. high MTP (HMTP) who received FFP: PRBC ratio ≥ 1:1.5 and low MTP (LMTP) who received a FFP: PRBC ratio of < 1:1.5. The ratio of FFP: PRBC administered was calculated for each patient at 4 h and the ratios were also calculated based on the blood products given from 4 to 24 h post injury in the same cohort to look for the effect of timing and transfusion ratios. Our analysis is based on the actual transfusion of blood products and MTP of our institution including 6 units of RBC, 6 units of FFP, and 6 units of platelets. Two units of uncross-matched blood were available to each patient without delay. All patients were followed-up until hospital discharge or death. The outcomes measures included mortality, 1^st^ MTP International Normalized Ratio (INR), multiorgan failure (MOF) and infectious complications.

We intended to analyze the outcomes in relation to the transfusion ratios calculated initially at 4 h and at 4–24 h. In particular, we looked at the effect of early (<4 h) MTP ratios on the final outcome.

### Definitions

The diagnosis of MOF was based on a maximum Marshall Multiple Organ Dysfunction score > 5. Ventilator associated pneumonia (VAP) diagnosis was based on the quantitative culture threshold of ≥10^4^ CFU/mL for broncho-alveolar lavage [11]. Blood stream infection (BSI) is a positive blood culture that not necessarily related to an indwelling central venous catheter. Whereas, line related infection was defined as catheter-related blood stream infections (CRBSI) which required positive peripheral cultures with the identical organism obtained from either a positive semi-quantitative culture (≥15 CFU/segment), or positive quantitative culture (≥10^3^ CFU/segment) from a catheter segment specimen. Urinary tract infection (UTI) was confirmed if the urine specimen revealed ≥10^5^ organisms/ml. This study was approved by the medical research center at Hamad Medical Corporation, Qatar (IRB # 11153/11).

### Statistical analysis

Data were presented as proportions, mean ± standard deviation (SD) or median as appropriate. Baseline demographic characteristics, clinical presentation, and outcomes were compared according to FFP: PRBC ratio at 4 h (HMTP *vs.* LMTP). Pearson chi-square (*X*^2^) test was used for categorical variables and student-*t* test for continuous variables. The Fisher’s exact test was used, if the expected cell frequencies were below 5. A significant difference was considered when the 2-tailed *p* value was less than 0.05. Data analysis was carried out using the Statistical Package for Social Sciences version 18 (SPSS Inc. USA).

## Results

During the 3- year study period, there were 4864 trauma admissions, of which 100 patients received massive blood transfusion. Finally, the study included 77 (1.6 %) cases in which ratios were attainable and the remaining 23 cases were excluded as they did not receive FFP within the first 4 h post-injury and so no ratio could be calculated. The mean age of patients was 33.7 ± 14 years and the majority of them were males (91 %). Blunt trauma accounted for most of cases (88.3 %). The median ISS, GCS and RTS were 29 (8–75), 4.4 ± 3.8, and 4 (1.2-7.8), respectively (Table 1). Lung contusion (62.3 %), head injury (50 %), pelvic (46.7 %) and rib fracture (35.5 %) were the most commonly associated injuries. The most frequent used operative interventions included exploratory laparatomy (66.2 %), Thoracotomy (24.7 %), repair of major vessels (22 %) and angioembolization (13 %). Among patients who underwent emergency operative interventions, 39 % had damage control surgery. The intra-operative mortality was 17 %. Focused assessment with sonography for trauma (FAST) was positive in more than half of the cases (53 %). The median intensive care unit (ICU) stay was 3 (1–45) days, the overall hospital length of stay was 2 (1–112) days and the hospital length of stay for patients who survived was 28 (8–112). Transport time to the trauma center was 64 ± 32.3 min. According to our institutional policy, blood transfusion begins only after arrival to the hospital, whereas prehospital crystalloid (normal saline) administration is initiated by EMS.

**Table 1:** Demographics and presentation of study cohort (n = 77)

Variable		Variable	
**Age** **(mean ± SD; years)**	33.7 ± 14	**Injured body region**	
**Males**	69 (90.8 %)	Head	32 (50 %)
**Blunt trauma**	68 (88.3 %)	Lung contusion	48 (62.3)
**Mechanism of injury**		Rib fracture	27 (35.5 %)
Traffic Pedestrian	22 (28.6 %)	Pelvic fracture	35 (46.7 %)
Traffic Driver	20 (26 %)	Long bone fracture	24 (32.0 %)
Traffic Passenger	12 (15.6 %)	Spleen	21 (27.3 %)
Fall From Height	10 (13 %)	Liver	25 (32.5 %)
fall of heavy object	5 (6.5 %)	Bowel	15 (19.5 %)
crush injury	1 (1.3 %)	Pancreas	9 (11.7 %)
Gunshot	1 (1.3 %)	Cardiac	4 (5.2 %)
Stab	5 (6.5 %)	**Operative interventions**	
**SBP (mmHg)**	85.2 ± 35.5	Exploratory Laparotomy	51 (66.2 %)
**Heart rate (b/min)**	119.3 ± 27.6	Thoracotomy	19 (24.7 %)
**Temperature in ED (°C)**	36.1 ± 0.66	Repair of major vessels	17 (22.1 %)
**FAST positive**	41 (53.2 %)	Chest tube insertion	7 (9.1 %)
**Laboratory Findings**		Craniotomy	1 (1.3 %)
Initial Hemoglobin (g/dL)	10.3 ± 2.6	External fixation	6 (7.8 %)
Initial Platelets (×10^9^/L)	187.7 ± 69.8	ORIF	1 (1.3 %)
Initial INR	1.6 ± 1.2	Mangled extremity amputation	3 (3.9 %)
Initial APTT (median)	30.3 (22–149)	Successful Angioembolization	10 (13 %)
Initial Fibrinogen (g/L)	0.96 ± 0.58	**Intra-operative status**	
Initial pH	7.1 ± 0.2	Stable + damage control	30 (39 %)
Initial HCO3 (mmol/L)	15.6 ± 3.8	Unstable + damage control	24 (31.2 %)
Saline	3739 ± 1411	Stable + procedure completed	8 (10.4 %)
Ringer Lactate (mmol/L)	1300 ± 958	unstable + procedure completed	1 (1.3 %)
**Blood products transfused <4 h**		**Intra-operative mortality**	13 (20.6 %)
PRBC	10.9 ± 4.9 [10 (2–23)]	**ICU LOS**	3 (1–45)
FFP	6.5 ± 3.8 [6(1–21)]	**Overall Hospital LOS (days)**	1.5 (1–112)
Platelet	6.4 ± 3.4 [6(1–22)]	**Hospital LOS**	28 (8–112)
**Severity Scores**		**Multi Organ Failure**	49 (63.6 %)
Injury Severity Score	29 (8–75)	**Overall mortality**	55 (71.4 %)
Revised Trauma Score	4 (1.2–7.8)		
Glasgow Coma Score	3 (3–15)		

*FFP* Fresh frozen plasma, *PRBC* packed red-blood-cells, *ICU* Intensive Care Unit, *LOS* length of stay, *FAST* Focused Assisted Sonography for Trauma, *INR* international normalized ratio, *APTT A*ctivated Partial Thromboplastin Time, *SBP* Systolic Blood Pressure, *ORIF* Open reduction internal fixation

bold means heading variable non-bold means subheadings of the main variable

Table 2 shows the clinical presentation and outcome of patients based on FFP: PRBC ratio (HMTP and LMTP) within the first 4 h. The mean age of the LMTP group was higher (37.6 ± 16.5 *vs.* 29.7 ± 9.5; *P* = 0.03) in comparison to HMTP group. However, both the HMTP (n = 31) and LMTP (n = 46) groups were comparable with respect to gender, mechanism of injury, prehospital intubation, thoracotomy, volume of normal saline administration, initial hemoglobin reading, INR, arterial pH and severity of injury (ISS & RTS). The incidence of hyperkalemia, hypomagnesemia and hypocalcemia were also comparable in the two groups. The mean arterial pH was higher among HMTP group post 1st MTP (7.2 ± 0.16 *vs.* 7.1 ± 0.23; *P* = 0.04) and 2nd MTP (7.3 ± 0.1 *vs.* 7.2 ± 0.2; *P* = 0.04) as compared to LMTP group. HMTP patients had better mean INR values after the 2nd MTP shipment (1.1 (1–3) *vs.* 1.5(1–3); *p* = 0.03) than LMTP. Significantly greater amount of Ringer lactate (1550 ± 1099 *vs.* 883 ± 431; *p* = 0.01) and combination of saline and Ringer lactate (4771 ± 1466 *vs.* 3883 ± 1580; *P* = 0.01) were transfused to patients with LMTP.

**Table 2:** Clinical presentation and complications based on high and low transfusion ratios within the first 4 h post-injury

	HMTP (n = 31)	LMTP(n = 46)	P value
Age (mean ± SD)	29.7 ± 9.5	37.6 ± 16.5	0.03
Blunt injury (%)	87.1	89.1	0.53
Penetrating injury (%)	12.9	10.9	
Saline (Prehospital)	1193 ± 666	1197 ± 737	0.97
Transport Time (min.)	64.4 ± 29.8	63.7 ± 34.3	0.93
Prehospital interventions			
Intubation	36 %	41 %	0.60
Thoracostomy	3 %	9 %	0.32
Laboratory findings			
Initial Hemoglobin (mean ± SD)	10.6 ± 3	10 ± 2.3	0.37
Initial INR	1.3 (1–10)	1.3 (1–3)	0.09
Initial pH	7.2 ± 0.14	7.1 ± 0.2	0.09
*1^ST^ MTP INR	1.35 (1–2)	1.7 (1–12)	0.09
1^st^ MTP pH	7.2 ± 0.16	7.1 ± 0.23	0.04
2^nd^ MTP INR	1.1 (1–3)	1.5 (1–3)	0.03
2^nd^ MTP pH	7.3 ± 0.1	7.2 ± 0.2	0.04
3rd MTP INR	1.1 (1–2)	1.2 (1–10)	0.17
3^rd^ MTP pH	7.2 ± 0.18	7.2 ± 0.2	0.55
Saline	3456 ± 1591	3929 ± 1258	0.15
Ringer Lactate	883 ± 431	1550 ± 1099	0.01
Saline + Ringer lactate	3883 ± 1580	4771 ± 1466	0.01
Cryoprecipitate	9.5 ± 0.7	8.4 ± 3.5	0.70
Calcium Gluconate/Chloride	1933 ± 1334	2250 ± 1674	0.54
Sodium Bicardonate	92.9 ± 73	152.3 ± 96	0.05
Fibrinogen	2800 ± 1095	3000 ± 1341	0.77
Factor VII	5.2 ± 2.4	3.8 ± 1.3	0.19
Injuries			
Head injury	52	48.6	0.80
Lung contusion	68	59	0.42
Rib fracture	47	28	0.10
Pelvic fracture	52	43	0.47
Long bone fracture	45	23	0.04
Spleen	19	33	0.2
Liver	26	37	0.3
Cardiac	0	9	0.09
Injury Severity Score	29.4 ± 11.6	32.5 ± 10.7	0.24
Revised Trauma Score	5.2 ± 2.3	5.2 ± 2.02	0.97
Glasgow Coma Score	8.5 ± 5	8 ± 5.1	0.72
Hyperkalemia (%)	17.2	29.3	0.19
Hypomagnesemia (%)	68.2	61	0.42
Hypocalcemia (%)	93	82	0.17
Complications			
Ventilator-associated Pneumonia (%)	32.3	14	0.05
Wound infection (%)	32.3	18.6	0.14
Bloodstream Infection (%)	6.5	11.6	0.37
CRBSI (%)	3.2	7	0.44
* Urinary tract infections* (%)	3.2	4.7	0.62
ACS (%)	6.5	2.2	0.35

*HMTP* high transfusion ratios, *LMTP* low transfusion ratios, *INR*, international normalized ratio, *ACS* abdominal compartment syndrome, *CRBSI* Catheter related Blood Stream Infection, *MTP* massive transfusion protocol

**Fig. 1:**
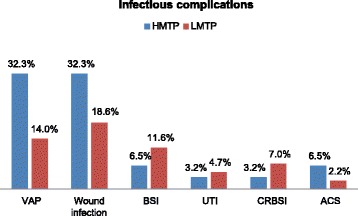
Distribution of infectious complications according to FFP: PRBC ratios

Though, the frequency of VAP, wound infection and abdominal compartment syndrome were higher in the HMTP group, these trends did not reach statistical significance. However, BSI and UTI were non-significantly higher in LMTP group (Fig. 1).

**Fig. 2:**
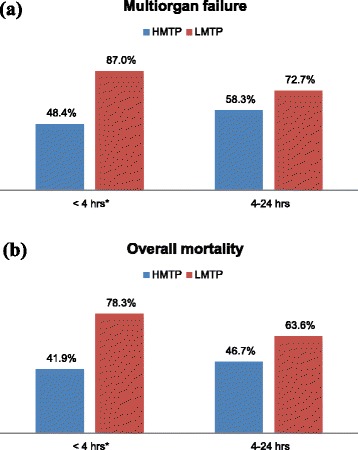
Outcome according to time post-injury (hours) and transfusion ratio (HMTP *vs.* LMTP) (**a**) multiorgan failure (MOF) (**b**) overall mortality

The overall mortality was 63.6 % (Table 3). Patients who died mainly had lung contusion (42.9 %), head injury (29.9 %), pelvic fracture (28.6 %), liver injury (24.7 %) and rib fractures (23.4 %). Moreover, significant number of patients in HMTP group who died had more rib fractures (66.7 % *vs.* 27.8 %; *P* = 0.01) than LMTP group. A non-significantly higher proportion of patients in LMTP group died within 6 h post-injury in comparison to HMTP. During the early period of resuscitation (<4 h), the incidence of MOF (48.4 % *vs.* 87.0 %, *p* = 0.001) and crude mortality (41.9 % *vs.*78.3 %, *p* = 0.001) were significantly lower in HMTP compared to LMTP group. However, in the later period (4–24 h) MOF and mortality were comparable among the two groups (Fig. 2).

**Table 3:** Outcome based on transfusion ratios

	HMTP (n = 31)	LMTP (n = 46)	*P* value
Mortality by associated injuries (ratios at *<4 h*)			
Head injury	88.9	55.6	0.07
Lung contusion	69.2	66.7	0.86
Rib fracture	66.7	27.8	0.01
Pelvic fracture	53.8	44	0.55
Long bone fracture	30.8	23.5	0.61
Spleen	15.4	27.8	0.37
Liver	30.8	41.7	0.48
Cardiac	0.0	8.3	0.28
Outcomes* based on MTP ratios calculated at <4 h			
Multi Organ Failure (%)	48.4	87	0.001
Mortality (%)	41.9	78.3	0.001
Outcomes* *based on MTP ratios calculated at* 4*–*24 h			
Multiorgan failure (%)	58.3	72.7	0.29
Mortality (%)	46.7	63.6	0.24

*It represents the overall outcome (MOF and mortality) and its correlation with the transfusion ratios calculated initially at 4 h and at 4-24 h

**Fig. 3:**
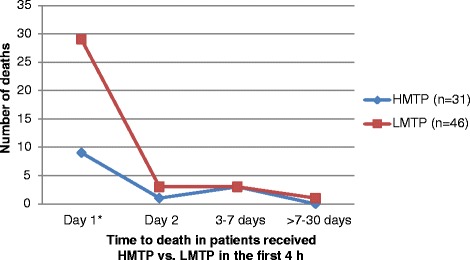
Number and time of hospital death in patients who received HMTP *vs.* LMTP in the first 4 h

Figure 3 demonstrates the time to all-cause mortality in those who received LMTP *vs.* HMPT (<4 h). The number of deaths was 3 times higher in LMTP in comparison to HMTP within the first 30 days (36 *vs.* 13 cases). The vast majority of deaths occurred within the first 24 h (80.5 % in LMTP and 69 % in HMTP) and particularly within the first 6 h (55 % *vs.* 46 %).

## Discussion

The present study evaluates the implication of FFP to PRBC ratio and its appropriate timing in trauma patients who required MTP. Our findings support the survival advantage of attaining high FFP to PRBC ratio for trauma patients who are identified early (within the first 4 h post-injury). Historically, Kashuk et al. [12] suggested a ‘bloody vicious cycle’ in which hemorrhage, cellular shock and tissue injury contributed to the formation of the lethal triad of coagulopathy, hypothermia, and acidosis. About 24 % of severely injured patients had acute traumatic coagulopathy on the hospital arrival [3]. Recent evidence suggests that coagulopathy should be thought of as a primary event, which is independently associated with detrimental outcomes. Therefore, more attention has been given to control the worse outcomes of acute traumatic coagulopathy through hemostatic resuscitation which improves patient outcomes [13]. Particularly, the role of FFP in managing traumatic coagulopathy is well documented. Despite the fact that use of high transfusion ratios is still a debatable issue, the majority of studies supported early and aggressive plasma use to attain FFP: PRBC transfusion ratios as high as 1:1 [4, 14, 15]. However, in the current practice there is no consensus on the optimal cut-off between adequately high and low plasma to PRBC ratios. Table 4 presents a comparison of several studies with respect to different FFP: PRBC cut-off ratios, timing and outcomes [4, 5, 14–24].

**Table 4:** Review of massive transfusion studies

Reference	N of pts	Mechanism	Massive transfusion definition	Time ratio calculated	Plasma : PRBC ratio	Results
Borgman et al., 2007 [4]	246	94 % penetrating (military)	≥9 U RBC in 1st 24 h	24 h	Low: 1:8,	Overall Mortality in low −65%, medium-34%, high-19 %, (*P* < .001).
					Medium: 1:2.5	
					High:1:1.4	
Sperry et al., 2008 [5]	415	100 % blunt	≥8 U RBC in 1st 8 h	12 h	Low: < 1 : 1.5	24-h mortality in low-12.8%, high - 3.9% (*P* = 0.012). Benefit gone by 48 h
					High: ≥ 1 : 1.5	
Holcomb et al., 2008 [14]	466	35 % penetrating	≥9 U RBC in 1st 24 h	24 h	Low: < 1 : 2	Improved 30-day survival in high ratio (59.6 *vs.* 40.4 %; *P* < 0.01)
					High: ≥ 1 : 2	
Gunter et al. 2008 (15)	259	55 % penetrating	≥10 U RBC in 1st 24 h	24 h	Low: < 1 : 1.5	reduction in 30-day mortality: high (41 %) *vs.* low (62 %) ratio group *p* = 0.008
					High: ≥ 1 : 1.5	
Maegele et al., 2008 [16]	713	92 % blunt	≥10 U RBC ED → ICU admission	NA	PRBC:FFP > 1.1, PRBC:FFP 0.9-1.1	6 h mortality 24.6, 9.6 & 3.5 % (*P* < 0.0001)
					PRBC:FFP < 0.9	24 h mortality 32.6, 16.7 & 11.3 % (*P* < 0.0001)
						30 day mortality 45.5, 35.1 & 24 % (*P* < 0.001)
Snyder et al., 2009 [17]	134	60 % penetrating	≥10 U RBC in 1st 24 h	24 h	Low: < 1 : 2	24-h mortality in low-58%, High - 40 %; but effect disappears when analyzed as time-dependent variable
					High: ≥ 1 : 2	
Teixeira et al., 2009 [18]	383	NA	≥10 U RBC in 1st 24 h	24 h	Low: ≤ 1:8,	Mortality rate decreased significantly with increased FFP; but effect disappears after a 1:3 ratio.
					Medium: >1:8 & ≤1:3	
					High: >1:3 & ≤ 1:2	
Mitra et al., 2010 [19]	331	86 % blunt	≥5 U RBC in 1st 4 h	4 h	>1:1.5	higher ratios were associated with significantly improved mortality rates
					>1:2.5 to 1:1.5	
					>1:3.5 to 1:2.5	
					≤1:3.5	
Magnotti et al., 2011 [20]	103	63 % blunt	≥10 U RBC in 1st 24 h	6 h	Low: < 1 : 2	6-h mortality was less in the high-group (10% *vs.* 48 %, *p* < 0.002)
					High: ≥ 1 : 2	
Lustenberger et al. 2011 [21]	229	100 % blunt	≥10 U RBC in 1st 24 h	12, 24 h	Low: < 1 : 1.5	High ratio was associated with improved survival at 12 and 24 h
					High: ≥ 1 : 1.5	
Brown et al., 2012 [22]	604	100 % blunt	≥10 U RBC in 1st 24 h	6, 12, 24 h	Low: < 1 : 1.5	High 6-h ratios were associated with a reduction in mortality risk at 6, 12, and 24 h (*p* < 0.05).
					High: ≥ 1 : 1.5	
Kudo et al., 2013 [23]	NA	NA	≥10 U RBC in 1st 24 h	6 h	High: >1:1.5,	Mortality rate: high (44.4 %);
					Medium: 1:1.5-1:2	Middle (16.7 %); low (33.3 %)
					Low: <1:2	
Holcomb et al., 2015 [24]	680	Severely injured (55 % blunt)	≥10 U of RBCs within 24 h	6 h	1:1:1 transfusion ratio of plasma, platelets, and RBCs to a 1:1:2 ratio	early administration of plasma, platelets, and red blood cells in a 1:1:1 ratio compared with a 1:1:2 ratio did not result in significant differences in mortality at 24 h or at 30 days
Present study	77	88.3 % Blunt	≥10 U RBC in 1st 24 h	4 h	Low: < 1 : 1.5	higher ratios were significantly associated with lower rate of mortality and MOF within initial 4 h of injury
		11.7 % penetrating			High: ≥ 1 : 1.5	

Our study revealed a significant improvement in the crude mortality and MOF rate when a FFP: PRBC transfusion ratio ≥1:1.5 was attainable within the initial 4 h of admission. As it have been shown in Fig. 3; the number of deaths within the first 30 days was 3 times higher in LMTP group and most deaths occurred within the first 24 h and particularly within the first 6 h in the 2 study groups.

Comparable survival benefit was documented in other studies that have used similar high transfusion ratios at different time durations [14–16]. However, achievement of such ratio as early as in the first 4 h and its impact on patient outcome has been described recently by Mitra et al. [19]. Consistent with our findings, the authors reported survival benefit for higher FFP: PRBC ratios during the first 4 h of resuscitation in severely injured blunt trauma patients. Moreover, the low FFP: PRBC ratio during the early phase was independently associated with the risk of mortality.

Of note, the improvement in mortality and MOF rates with high transfusion ratios were observed only during the early period of hemostatic resuscitation and were not seen later on (>4 – 24 h) in our study. Hence, we believe that the survival benefit observed in other studies when the ratios were calculated at 24 h is possibly a dilution effect.

Snyder et al. [17] introduced the question of survival bias for transfusion ratio in massively transfused trauma patients. The authors observed a significant association between improved survival and high transfusion ratios during the first 24 h. But this relationship did not sustain after adjustment for survival bias in the study cohort.

Similarly, Teixeira et al. [18] conducted a 6-year retrospective study to analyze the effect of plasma transfusion (≥10 PRBC) among massively transfused patients and observed a survival benefit for higher FFP: PRBC ratio during initial 24 h. De Baisi et al. [25] reported that mortality was significantly correlated with worse plasma deficit during the initial 2 h of resuscitation but no association with plasma ratio was reported. A similar association was observed among the massive transfusion group at 24 h.

Several studies have shown a correlation between FFP transfusion and the development of hospital complications. Khan et al. [26] suggested that FFP transfusion is independently associated with the risk of acute lung injury and acute respiratory distress syndrome (ARDS). In addition, Sperry et al. [5] observed 2 times higher risk of ARDS in patients resuscitated with a high FFP: PRBC ratio. In contrast, our study reported a lower MOF rate among the high ratio group within the early as well as late phase. Both groups were comparable in terms of the initial coagulopathy, injury severity and demographic status except for age. We also noted progressive improvement in the coagulopathy status in the high ratio group. These results substantiate our current understandings of the utility of aggressive plasma use to prevent and treat early onset coagulopathy after injury. Furthermore, the rate of nosocomial infections did not reach statistical significance in either group. However, high ratio group patients had higher rates of wound infection and pneumonia which might explain the potential complications and risks attributable to this type of resuscitative practice.

Our study has some limitations including its retrospective nature. Being a single institution study, we are limited with the small sample size and power in addition to the lack of detailed description of the coagulation management. We were also not able to account for the incidence of ARDS. Also, the possibility of survival bias and incompleteness of collected data cannot be ruled out. We were looking for the MTP and the death burden from exsanguination, not the over-all death burden. After all, an MTP’s main objective is to reduce deaths from traumatic hemorrhage. All deaths due to the severity of injury were ‘pre-ordained’ already. To date most of the studies have calculated FFP: PRBC ratios at 24 h. Therefore, analysis of ratios at 4 h would help to counter survival bias to some extent [19]. The proper ratios and time for administrating MTP in trauma patients are not well defined yet in the literatures (Table 4). In our institute, the MTP protocol was fully implemented in 2011 and so the difference in transfusion practice can be attributed to practice variation as well as the ‘learning curve’ that attends the implementation of any new clinical protocol. Furthermore the data were collected retrospectively so we cannot assume the exact reason behind this time and ratio variations except for physician discretion in the absence of hospital protocol at that time. Lastly, multi-year retrospective studies might suffer from the same ‘potential bias’ that could be encountered in our analysis.

## Conclusions

The mortality risk associated with low FFP: PRBC ratios of <1:1.5 may occur very early, possibly secondary to ongoing coagulopathy and hemorrhage. Aggressive attainment of high FFP/PRBC ratios as early as 4 h post injury can substantially improve coagulopathy and reduce mortality and MOF rates. The present study is an audit of massive transfusion strategies used at our center which highlights the current experience of managing exsanguinating trauma patients. The analysis of appropriate transfusion ratios and timings provides useful information regarding the correct ratios of blood component and avoiding wastage of the blood products. This information can form the basis for developing research based uniform massive transfusion guidelines for the appropriate use of various blood components. Furthermore, it can be the basis for designing massive transfusion research focusing high transfusion ratios targeted within the first 4 h. Therefore, large prospective studies are needed to validate our findings for a balanced FFP: PRBC ratio that would decrease the overall PRBC utilization and the decision needed for massive transfusion.

## Abbreviations

MTP: Massive transfusion protocol
MOF: multiorgan failure
FFP: fresh frozen plasma
PRBC: Packed red blood cells
ISS: Injury Severity Score
RTS: Revised Trauma Score
HMTP: high MTP
LMTP: low MTP
INR: International Normalized Ratio
VAP: Ventilator associated pneumonia
BSI: blood stream infection
CRBSI: Catheter-related blood stream infections
UTI: Urinary tract infection
